# Comparison of the Minimally Invasive Reverdin–Isham Lateral Translation Osteotomy Versus the Standard Reverdin–Isham Technique: A Pilot Prospective Cohort Study

**DOI:** 10.3390/jcm13185468

**Published:** 2024-09-14

**Authors:** Maria Belda-Donat, Luis M. Marti-Martinez, Rubén Lorca-Gutierrez, Carmen Naranjo-Ruiz, Fernando Chacón-Giráldez, Carlos Barrios

**Affiliations:** 1 School of Doctorate, Valencia Catholic University “San Vicente Mártir”, 46001 Valencia, Spain; mariabeldadonat@gmail.com; 2 Behavioural and Health Sciences Department, Miguel Hernandez University, 03550 San Juan de Alicante, Spain; 3 Physiotherapy and Podiatry Department, Valencia Catholic University “San Vicente Mártir”, 46001 Valencia, Spain; 4 Podiatry Department, Faculty of Medicine and Health Sciences, Valencia Catholic University “San Vicente Mártir”, 46001 Valencia, Spain; carmen.naranjo@ucv.es; 5 Podiatry Department, Faculty of Nursing, Physiotherapy, and Podiatry, Universidad de Sevilla, 41009 Sevilla, Spain; 6 Institute for Research on Musculoskeletal Disorders, Valencia Catholic University “San Vicente Mártir”, 46001 Valencia, Spain; carlos.barrios@ucv.es

**Keywords:** hallux valgus, percutaneous surgery, lateral translation technique

## Abstract

**Background/Objectives:** Reverdin–Isham osteotomy is effective in correcting moderate hallux valgus deformity but has certain limitations when correcting a deformity in the sagittal plane. This study aimed to evaluate the impact on pain, functionality, and radiological measures of angular corrections, and the safety of the Reverdin–Isham lateral translation technique through minimally invasive surgery in the treatment of a moderate hallux valgus compared to Reverdin–Isham standard osteotomy. **Methods:** A pilot 6-month prospective cohort study was conducted on adults over 18 years old with a hallux valgus in at least one foot. The study exposure was the use of the Reverdin–Isham lateral translation technique. The outcome variables were pain and functionality through VAS and AOFAS scales, respectively, and radiological measurements of the first toe metatarsophalangeal angle (MPA), first space intermetatarsal angle (IMA), proximal articular set angle (PASA), distal articular set angle (DASA), metatarsal formula, and position of sesamoids in the AP projection. **Results:** The study involved 60 participants. Results indicate significant reductions in pain and radiological measures in both cohorts: MPA improved by 23.13 degrees, IMA by 5.93 degrees, and sesamoid position by 4.23 degrees in patients who underwent the lateral translation technique versus 13.20, 3.30, and 1.57 degrees, respectively, in patients who experienced the standard Reverdin–Isham technique. The lateral translation method showed greater reductions in these metrics compared to the standard Reverdin–Isham technique (*p* < 0.05). **Conclusions:** Percutaneous Reverdin–Isham techniques, both standard and with lateral translations, effectively corrected moderate hallux valguses. However, the lateral translation method provided greater reductions in MPA, IMA, and sesamoid positions, making it more suitable for deformities with IMAs over 15 degrees.

## 1. Introduction

The hallux valgus (HV) is a frequent, complex, and progressive deformity of the alignment of the first metatarsophalangeal joint (MTPJ) due to the multifactorial etiology. The average prevalence of hallux valgus in the general population is 19%, increasing to 22.7% in adults over 60 years old. This condition affects up to 23.7% of adult women in contrast to 11.4% in men [1].

Different surgical techniques have been described to address HV treatment, most of which involve osteotomy of the first metatarsal bone. The osteotomy can be performed in different areas of the first metatarsal, either in its distal, middle, and/or proximal bone areas, accompanied or not by the release of surrounding soft tissues [2,3]. In recent decades, percutaneous techniques for HV treatment have gained popularity, based on the premises of a shorter surgical time, reduced surgical trauma, reduced postoperative pain, and faster recovery [4]. The percutaneous procedure for moderate HV correction evolved from the traditional open technique of Kramer [5]. In 1990, Bösch et al. [6] modified Kramer’s osteotomy by performing the procedure through a minimal incision using a high-frequency reamer. He presented his percutaneous technique detailing a lateral cephalic displacement equivalent to 75% of the minimum metatarsal diameter, achieving a consolidation rate of 100% using a Kirschner wire to secure the osteotomy [7].

One of the most commonly used percutaneous procedures, the Reverdin–Isham osteotomy, emerged in 1991, and has proven to be effective in correcting moderate HV deformity [7,8]. However, this technique has certain limitations in correcting deformities in the sagittal plane. Furthermore, when it is necessary to correct the intermetatarsal angle (IMA), the Reverdin–Isham procedure requires complete lateral cortical osteotomy to allow the metatarsal head to shift laterally. This surgical gesture conforms to the Reverdin–Isham technique with lateral translation, which is based on the minimally invasive surgery (MIS) modified by Bösch [6,9]. In Spain, this modified technique is already being used in some private podiatry centers. There is a general agreement in the literature concerning clinical and patient satisfaction results provided by percutaneous techniques, which have been found similar or even superior to those observed with open techniques [10]. However, despite the findings reported in these studies, there still remains a debate and controversy regarding the follow-up time and the level of evidence (III and IV) associated with MIS [11,12].

Therefore, although forefoot percutaneous surgery has gained interest among foot surgeons, uncertainty remains as to which specific technique within minimally invasive surgery is the most effective. The lack of direct comparative studies between various percutaneous techniques, beyond systematic reviews [13], represents a notable gap in the scientific literature. In the current study, a comprehensive analysis and detailed comparison between two percutaneous distal metatarsal osteotomies was conducted, aiming to address this knowledge gap. The hypothesis of this study was that the Reverdin–Isham technique with lateral translation by MIS, in patients with moderate HV, improves pain, radiographic angles, functionality, and postoperative complications compared to the group of patients undergoing the standard Reverdin–Isham technique also by MIS. Thus, the main objective of this study was to evaluate the impact, in terms of pain, functionality, and radiological measures of angular corrections, and safety of the Reverdin–Isham lateral translation technique through MIS in the treatment of moderate HV compared to the Reverdin–Isham standard osteotomy.

## 2. Materials and Methods

### 2.1. Study Design and Setting

A pilot 6-month prospective cohort study was conducted between 2021 and 2023 in five private podiatric centers in Spain (one center in Madrid, two centers in Valencia, and two centers in Alicante), including two cohorts of patients. In the center located in Madrid, the podiatrist used the standard Reverdin–Isham technique routinely, while the podiatrists in the rest of the centers had already begun applying the modified technique some time before designing and initiating this study. All the podiatric surgeons involved in this study had more than 15 years of clinical and surgical experience. They were all faculty members at various universities and official master’s programs, and instructors in minimally invasive foot surgery workshops.

### 2.2. Inclusion and Exclusion Criteria

Patients of both sexes, aged 18 and over, with a diagnosis of moderate HV in at least one foot, who attended treatment at the participating podiatry centers, and had not responded satisfactorily to conservative treatment for at least one year were eligible for the study. The identification of moderate HV in this study was based on the Coughlin and Mann classification [14], which is widely used in orthopedics. This classification uses radiographic criteria, specifically the intermetatarsal and metatarsophalangeal angles, to determine whether hallux valgus is mild, moderate, or severe. Subject recruitment was conducted from November 2021 to June 2022 by a collaborating podiatrist at each center, using consecutive sampling.

All participants who voluntarily agreed to be part of the study, providing informed consent to undergo surgical intervention and participate in the study, and who were also available for follow up after the surgery, were included in the study. Patients who had previously undergone foot surgeries, as well as those with psychological disorders, degenerative diseases, neuromuscular conditions, osteoarticular or musculoligamentous conditions, and other forefoot deformities, such as hallux rigidus, digital supraductus, or claw toes, were excluded from the study.

One cohort consisted of patients from the centers utilizing the Reverdin–Isham with lateral translation (RIT) osteotomy, while the other cohort comprised patients from the center where the conventional Reverdin–Isham (RI) osteotomy was still being performed. Both techniques were conducted using MIS.

### 2.3. Study Variables

The study variables were age, sex, participating center, foot with moderate HV (right/left), bunion (yes/no), paresthesia (yes/no), pain on pressure (yes/no), altered metatarsophalangeal mobility (yes/no), transfer of central metatarsal load (yes/no), metatarsal index (minus/plus/plus minus), type of surgery received (RIT/RI osteotomy), pain measured using the VAS scale (Visual Analog Scale) and AOFAS (American Orthopaedic Foot and Ankle Society) scale, foot functionality (AOFAS scale), toe alignment with HV (AOFAS scale), clinical and functional status (AOFAS scale), radiological measurements in anteroposterior projection, and surgery complications. All information was collected by the collaborating podiatrist at each center in the study case report form, and radiographs were requested at a specialized center external to the podiatry clinic.

#### 2.3.1. Outcome Measures

Pain: measured using the VAS, which assesses pain intensity on a continuous scale typically from 0 to 10. Additionally, the AOFAS scale was used, specifically its pain dimension, to further evaluate pain severity.

Functionality: each patient underwent a preoperative and postoperative physical examination of the feet and completed the AOFAS. The AOFAS scale addresses subjective aspects such as pain, categorized into severity levels (severe, moderate, mild, or absent), as well as functionality (from severe limitations in daily activities to no limitations). These subjective values were summed up to a maximum of 60 points and, combined with the examiner’s objective evaluation (40 points), provided a measure of the patient’s outcome (100 points) in terms of digital alignment, mobility, and stability [15]. Scores ranging from 90 to 100 were considered excellent, 72 to 89 as good, 41 to 71 as fair, and below 40 as poor. Scores of 91 or higher may indicate the absence of pathology in individuals, which could be considered a threshold for determining total satisfaction with surgical treatment [16,17].

Radiological evaluation: preoperative and postoperative assessments for all patients included dorsoplantar (AP) and lateral weight-bearing foot radiographs. Measurements were taken of the first toe metatarsophalangeal angle (MPA), first space intermetatarsal angle (IMA), proximal articular set angle (PASA), distal articular set angle (DASA), metatarsal formula, and position of sesamoids in the AP projection. In the lateral radiographic view, the relative inclination of the dorsal cortex of the first metatarsal to the dorsal cortex of the second metatarsal was recorded.

Safety: any complications during and after the surgery was recorded. Intraoperative complications included issues such as excessive bleeding, nerve damage, or fractures. Postoperative complications, recorded at six months of follow up, encompassed a range of issues including cellulitis, persistent edema, anteroposterior misalignment, range of motion (ROM) limitations in the first metatarsophalangeal joint, and neuritis at the incision site. Complications were documented by the collaborating podiatrist at each follow-up visit, with a focus on identifying any cases of hallux varus due to overcorrection, defective or delayed consolidation, pseudoarthrosis, thromboembolism, infections (superficial or deep), avascular necrosis of the metatarsal head, and dorsal displacement of the metatarsal head. These complications were assessed through a clinical examination and radiological evaluation, ensuring a comprehensive analysis of the safety profile of each surgical technique.

#### 2.3.2. Exposure: Surgical Techniques

All patients underwent outpatient surgery. The anesthetic protocol consisted of a 2% mepivacaine nerve block as none of the patients were allergic to this drug. A 2 mm lateral incision was made at the distal metaphysis of the first metatarsal using a #15 scalpel blade (Bard-Parker, Aspen Surgical, Caledonia, MI, USA) until reaching the bone. Subsequently, the dorsal and plantar areas were deperiostealized at the site of osteotomy and dorsolateral exostosis. A cylindrical burr was then inserted to perform bunionectomy connected to the reducing handpiece and Osada Success 40 micromotor (Osada Electric Co., Ltd., Tokyo, Japan) and SH-29 handpiece (Osada Electric Co., Ltd., Tokyo Japan). Residual bone debris was removed using a surgical file. Subsequently, through the same incision, the Reverdin–Isham osteotomy procedure was performed.

**Figure 1:**
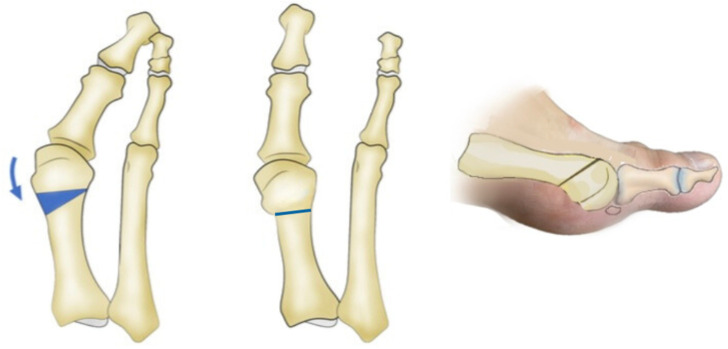
Standard Reverdin–Isham osteotomy.

**Figure 2:**
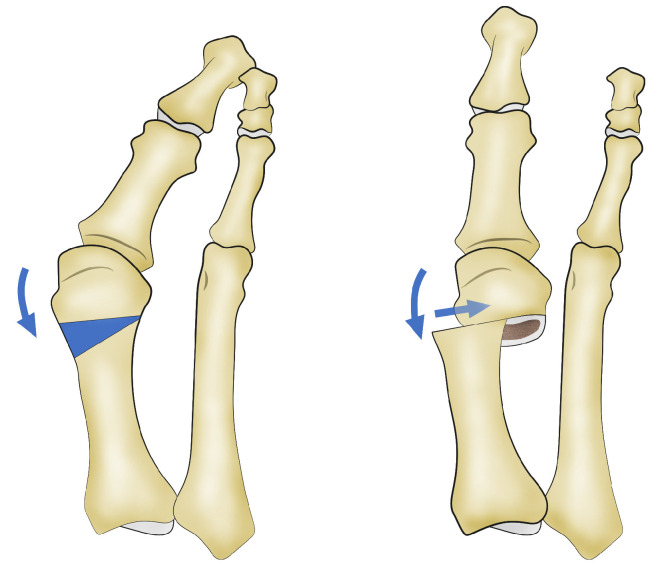
Reverdin–Isham osteotomy with lateral translation of the first metatarsal head.

- The standard Reverdin–Isham osteotomy procedure was performed using the Shannon–Isham long burr perpendicular to the axis of the first metatarsal in the sagittal plane [18,19]. The mediolateral inclination in the frontal plane determines the degree of metatarsal shortening. Notably, the lateral cortex of the metatarsal remains preserved (Figure 1). This was accompanied by an adductor tenotomy through a perpendicular incision made with a Beaver 64 knife (Beaver-Visitec International, USA) using fluoroscopy to locate the lateral sesamoid, deepening until locating the adductor muscle. The stabilization of the osteotomy was achieved with a bandage that aligned the metatarsophalangeal joint and with post-surgical footwear, without using osteosynthesis material.
- The Reverdin–Isham lateral translation technique was performed by conducting the standard Reverdin–Isham osteotomy as described above, with the particularity of sectioning the lateral cortex so that the osteotomy of the metatarsal was complete, allowing for the lateral displacement of the metatarsal head toward the inside of the foot. A tenotomy of the adductor, a lateral capsulotomy, and the release of the collateral and suspensory ligaments were performed using the Beaver 64 scalpel (Beaver-Visitec International, Waltham, MA, USA) and a fluoroscope to locate the adductor muscle, following the same procedure as described above (Supplementary Figure S1). In this cohort, the capsulotomy was added through the same incision, locating first the metatarsophalangeal joint interline and making an incision of the dorsolateral phalangeal capsule. This procedure allows the lateral displacement of the head of the first metatarsal (Figure 2, Figure S2 and Figure S3). In cases of severe hallux valgus (Supplementary Figure S4), characterized by the valgus rotation of the metatarsal and a convex lateral cortex (Supplementary Figure S5), the osteotomy is indicated with a shortening design to allow a greater translation of the metatarsal head. This results in a concave lateral cortex and better correction of the intermetatarsal angle. This osteotomy is always accompanied by soft tissue release, as previously described (Supplementary Figure S6). Supplementary Figure S7 shows the anteroposterior view and the weight-bearing anteroposterior X-ray of the right foot with moderate hallux valgus from a patient who underwent a modified Reverdin–Isham osteotomy with lateral translation, before surgery and 6 months after surgery.

Following the Reverdin–Isham osteotomy, with or without translation of the metatarsal head, a conventional Akin osteotomy was performed through a perpendicular incision in the proximal metaphysis of the proximal phalanx of the hallux with a Beaver 64 knife. The incision was perpendicular to the skin until reaching bone, and a blunt elevator and file were used to mark the location of the osteotomy. A long Shannon burr was then introduced perpendicular to the longitudinal axis of the proximal phalanx of the hallux controlled by fluoroscopy. The surgical procedure was finalized by verifying the surgical procedures and alignment of the hallux using fluoroscopy. The incision was closed with a single discontinuous suture of 4/0 nylon monofilament (Ethicon Inc., Raritan, NJ, USA). A specific dressing with Hypafix non-woven tape strips (BSN medical GmbH, Hamburg, Germany) was applied to maintain osteotomy fixation, without using osteosynthesis material in any of the osteotomies, followed by cohesive bandage wrapping. It should be noted that after the Reverdin–Isham osteotomy with lateral translation, during the first week, a hypercorrected position of the first ray occurs, where the base of the proximal phalanx acts as a fulcrum to help stabilize the metatarsal head in the desired displacement (Supplementary Figure S8). Over the next six weeks, the alignment of the first ray is adjusted to the proper position, similar to the technique described earlier for the Reverdin–Isham procedure (Supplementary Figure S9). All patients were provided with a post-surgical shoe with a rigid sole and full support during the recovery period, allowing them to walk.

### 2.4. Follow Up

Postoperative follow up was conducted according to established professional criteria. The first fluoroscopy follow up was performed 72 h after surgery, followed by weekly visits for dressing changes for 4–6 weeks until confirming osteotomy consolidation under fluoroscopy. During these standard follow-up visits, only complications were recorded for the study. Six months after surgery, patients were asked to return to the center where the surgical procedure was performed to complete their AOFAS scale measurements and radiological evaluation. At this final visit, all the information on the outcome variables (pain, functionality, radiological evaluation, and complications) was collected. These follow-up periods were chosen considering the time required for gait normalization after the disappearance of residual edema and any type of pain associated with the intervention. Patients did not undergo any postoperative physiotherapy programs or receive any additional treatment.

### 2.5. Sample Size

Sample size calculation was not performed as this study was a pilot study. Two cohorts were formed, each consisting of 30 patients.

### 2.6. Statistical Analysis

A descriptive analysis of all variables was performed by calculating frequencies for qualitative variables and minimum, maximum, mean, standard deviation, and median with interquartile range for quantitative variables. Normality of the variables was checked using the Kolmogorov–Smirnov test. The 95% confidence intervals for the means were calculated using a bootstrap procedure with 1000 simulations. Homogeneity of the cohorts with baseline variables was checked using Fisher’s test or the Mann–Whitney U test, as appropriate. The mean change in each variable before–after in each cohort was evaluated using the Wilcoxon test. To compare the mean changes before–after between groups, Delta variables were constructed as the difference in values between after minus before, such that negative values indicate a decrease, and positive values indicate an increase. Values close to zero indicate no changes before–after. Mean values of Delta variables between cohorts were compared using the Mann–Whitney U test. Due to the differences between subjects in the two cohorts, there may be potentially confounding variables affecting the observed change between groups, and to control for this, multivariate linear models were adjusted to each Delta variable, taking the cohort as the study factor, and adjusting variables showing differences between groups. Normality of Delta variables was assessed, performing both crude and multivariate adjustment. As a measure of fit, the adjusted R2 in percentage of each multivariate model was calculated. The analyses were performed using SPSS v.28 and R v.4.3.1 software.

## 3. Results

The whole sample consisted of 60 participants (6 men and 54 women) with a mean age of 58.3 (SD13.2) years, with 17 patients from two centers in Valencia, 13 from two centers in Alicante, and 30 from the center in Madrid. The cohort undergoing the standard Reverdin–Isham technique has a lower mean age than the patients in the cohort who underwent the Reverdin–Isham technique with metatarsal head translation but without a significant statistical difference (55.1; SD 12.7 versus 61.4; SD 13.1; *p* = 0.072). The discrepancy in the number of male and female participants was incidental, resulting from the consecutive recruitment of eligible patients for surgical treatment according to their admission to the study centers and the higher incidence of HV among females. Patients’ clinical profiles are summarized in Table 1. The statistically significant differences between the two cohorts were the higher proportion of patients with pressure pain in the RI cohort (96.7% versus 80%; *p* = 0.044), the lower proportion of patients of the standard RI cohort with load transfer to central metatarsals compared to patients of the RIT cohort (43% versus 90%; *p* < 0.001), and the proportion of Index Minus and Index Plus is significantly higher in the RI cohort than in the RIT cohort, while Index Minus Plus is lower in the RI cohort than in the RIT cohort for the pre-metatarsal formula variable (*p* = 0.028).

Table 2 shows the score of pain, total AOFAS, and radiological measurements corresponding to the two cohorts before and after surgery, and Supplementary Table S1 shows the changes in each AOFAS domain before and after surgery in both groups. Patients operated on by Reverdin–Isham technique with a lateral translation of the metatarsal head expressed more pain according to the VAS scale and lower scores in the AOFAS pain domain, also indicating more subjective pain. The differences between the two cohorts were statistically significant. The RIT cohort also exhibited a greater misalignment of the hallux that was reflected in the lower scores in the corresponding domain. AOFAS function scores were almost similar in the two groups. There were also statistically significant differences between the two cohorts in all radiological measurements. HV deformity in patients operated on adding metatarsal head translations was more severe, having higher MPA, IMA, PASA, and DASA. The position of the sesamoids of the first toe presented higher subluxation in patients of the cohort in which the Reverdin–Isham technique was performed with a metatarsal head translation, and the differences were also statistically significant compared to the cohort receiving the conventional technique.

Significant changes were observed in all variables before and after, in both groups, except for IF mobility in the RIT group (Supplementary Table S1). Delta variables were analyzed to assess whether these changes occurred differently in each group (Supplementary Table S2). The reduction in the mean values of MF1, IM, and sesamoid position after surgery was significantly greater in the RIT group compared to the RI group (Table 2). However, there was no significant difference in the mean change in pain (VAS), AOFAS score, PASA, and DASA between both groups.

As no large deviations from a normal distribution were observed for any Delta variable in each group (Supplementary Table S3), linear models were fitted taking the Delta variables as the dependent variables, and adjusting for the age and the two potentially confounding variables of the effect on the response variables identified in the analysis of homogeneity between groups (Table 1): pressure pain and load transfer to central metatarsals. The crude adjustment again showed a significantly greater decrease in the variables MF1, MI, and sesamoid position in the RIT group compared to the RI group from before to after the intervention. After performing the multivariate adjustment, these differences were attenuated, as some of the variations could be attributed to the heterogeneity of the groups regarding the adjustment variables. Nevertheless, the RIT group still showed a significantly greater reduction in MF1, MI, and sesamoid position (Table 3).

During the study, no intraoperative complications were evident in any of the investigated groups. However, it is noteworthy that five cases of postoperative complications were observed in each group. In the group subjected to the Reverdin–Isham technique with lateral translation, one case of cellulitis, one case of persistent edema, one case of anteroposterior misalignment, and two cases of mild stiffness in the range of motion (ROM) of the first metatarsophalangeal joint (30–70 degrees) were recorded. In the group treated with the standard Reverdin–Isham technique, two cases of persistent edema, two cases of limitation in the ROM of the first metatarsophalangeal joint, and one case of neuritis at the incision of the proximal phalanx of the first toe due to a hypertrophic scar. This case was resolved by surgical intervention for neural release.

It is important to note that no cases of hallux varus due to overcorrection, defective consolidation, delayed consolidation, or pseudoarthrosis were recorded in any of the studied groups. Additionally, no serious complications, such as thromboembolism, superficial or deep infections, or avascular necrosis of the metatarsal head, occurred. It is important to highlight that no dorsal displacement of the metatarsal head was observed in any case throughout the study.

## 4. Discussion

The present study showed that the surgical correction of HV was effective in both the Reverdin–Isham technique (RI) and the Reverdin–Isham technique with lateral translation (RIT) cohorts, as statistically significant improvements were observed from preoperative to postoperative assessments in all clinical and radiological variables. Both techniques resulted in relevant pain reduction as measured by the VAS and total AOFAS scores, with no significant differences between the two cohorts in terms of pain relief. Notably, the RIT cohort exhibited a greater and statistically significant correction of the MPA, IMA, and sesamoid position compared to the standard RI cohort. Both techniques provided similar significant corrections in the PASA and DASA measures.

Portaluri [20] and Magnan et al. [21] reported case series including 118 feet treated with the subcutaneous Bösch technique without screws, demonstrating that the clinical outcomes obtained were comparable to those of conventional open surgery. Giannini et al. [22] conducted a randomized study comparing the clinical–radiological outcomes of 40 patients with bilateral hallux valguses, treating one foot with scarf open osteotomy and the other with Bösch percutaneous surgery without screws. Although they did not find statistically significant differences in postoperative angular measurements, they observed that percutaneous surgery reduced surgical times, suggesting an efficiency advantage.

The results of the present study demonstrate notable improvements in MPA, IMA, and AOFAS scores for both the Reverdin–Isham technique and its lateral translation variant, aligning with prior systematic reviews (Table 4).

While previous studies, such as those by Malagelada et al. [13] and Bia et al. [23], report similar trends in radiological and clinical outcomes, our findings suggest a greater correction in MPA and IMA, particularly with the lateral translation variant. This could be attributed to the enhanced ability of the lateral translation to address larger deformities, similar to the Bösch technique, which allows for complete metatarsal head translation. In contrast, the medial closing wedge osteotomy in the standard Reverdin–Isham procedure seems to have a more limited capacity for reducing IMA. Bia et al. [23] concluded that better post-surgical clinical results were obtained on the AOFAS scale with the Reverdin–Isham technique, while the Bösch technique achieved better radiological correction. Our findings mainly agree with the results of this latter study since the AOFAS scale values in the Reverdin–Isham cohort are slightly higher, and the radiological corrections are more significant in the lateral translation Reverdin–Isham cohort. The consistency in AOFAS score improvements across studies highlights the overall effectiveness of both techniques in improving function and reducing pain, though our study shows a slightly higher functional gain, likely due to the focus on moderate deformities.

Regarding complications, the addition of the lateral translation of the metatarsal head to the Reverdin–Isham osteotomy did not result in a higher incidence of complications. Both the standard technique and the variant with a metatarsal head translation exhibited a similarly low percentage of postoperative complications (16.6%) and all of them were considered as minor complications. These complications did not significantly affect the final clinical outcomes as assessed by the AOFAS. Only one patient required minor surgery for the neurolysis of a digital nerve entrapped within postoperative scar tissue. In the present study, the complication rate was much lower than that reported in previous studies regarding Reverdin–Isham osteotomy. Biz et al. [19] recorded complications in 31.25% of patients in a longitudinal prospective study of 80 cases operated on using Reverdin–Isham and Akin percutaneous osteotomies at a 48-month follow up. These authors found 7.5% of major complications (five patients with HV recurrence and one with severe stiffness of the metatarsophalangeal joint) and 23.75% of minor complications, all (16 cases) showing a slight reduction in the normal range of MTP joint motion (ROM 30–74°). Stiffness of the first MTP is one of the most commonly described side effects of Reverdin–Isham percutaneous osteotomy, as it is an intra-articular medial closing wedge osteotomy [25,26]. However, only two cases with a limited range of motion were identified in each series (6.6%). In accordance with Bauer et al. [27], an adequate and frequent lavage during HV surgery, particularly after bunionectomy, can effectively prevent this complication.

Since the Reverdin–Isham technique does not involve fixation, concerns arise about osteotomy stability, potentially leading to delayed healing, non-union, and loss of correction. However, in our series, no instances of delayed unions or nonunion were observed. There was only one case of loss of alignment during the initial postoperative period, which occurred in the Reverdin–Isham group with metatarsal translations. It appears that the slight oblique Reverdin–Isham osteotomy (from dorsal to ventral) provides sufficient stability for weight-bearing, allowing the metatarsal head to attain its correct position.

One limitation of the study could be the low number of patients recruited in each cohort. However, since this is a pilot study, the number of participants can be considered adequate to obtain promising preliminary results. Pilot studies generally do not require specific sample size calculations. Another limitation is that the follow-up data were restricted to 6 months after surgery. This limitation highlights the need to continue following up these patients to evaluate longer-term outcomes. Additionally, the study had a low representation of male patients, indicating the necessity for further research focusing on this sex. Although there is some controversy among authors regarding the use of the AOFAS scale [28,29], it remains a widely used clinical tool among foot surgeons [30,31]. In this study, the AOFAS scale was split into its individual components of pain, functionality, and alignment, with the aim of investigating the possible relationship between the different surgical techniques studied and improvements in outcomes following intervention.

## 5. Conclusions

Percutaneous techniques of Reverdin–Isham with a lateral translation and the standard Reverdin–Isham proved to be effective in correcting percutaneously moderate hallux valguses, achieving the adequate correction of deformities, with additional benefits, such as shorter surgical times and reduced invasiveness. Notably, the Reverdin–Isham lateral translation technique demonstrated superior outcomes, with greater reductions in MPA, IMA, and sesamoid position compared to the standard technique. This suggests that the lateral translation variant may be more suitable for cases with more severe deformities, particularly when the IMA exceeds 15 degrees. These findings support the efficacy of both techniques, while highlighting the advantage of lateral translation in achieving greater deformity correction. Future research should continue to investigate and compare different percutaneous approaches to optimize outcomes for a broader range of forefoot deformities and patient profiles.

## Figures and Tables

**Table 1:** Baseline characteristics of the study participants and analysis of the homogeneity of the cohorts.

		Total Sample n = 60	RIT Cohort n = 30	RI Cohort n = 30	
		n (%)	n (%)	n (%)	*p*-Value ^a^
Sex	Male	6 (10.0)	2 (6.7)	4 (13.3)	0.671
	Female	54 (90.0)	28 (93.3)	26 (86.7)	
Foot	Right	29 (48.3)	15 (50)	14 (46.7)	0.796
	Left	31 (51.7)	15 (50)	16 (53.3)	
Bunion	No	3 (5.0)	2 (6.7)	1 (3.3)	1.000
	Yes	57 (95.0)	28 (93.3)	29 (96.7)	
Paresthesia	No	40 (66.7)	22 (73.3)	18 (60)	0.273
	Yes	20 (33.3)	8 (26.7)	12 (40)	
Pressure pain	No	7 (11.7)	6 (20)	1 (3.3)	0.044
	Yes	53 (88.3)	24 (80)	29 (96.7)	
Altered MTF mobility	No	9 (15.0)	7 (23.3)	2 (6.7)	0.145
	Yes	51 (85.0	23 (76.7)	28 (93.3)	
Transfer of central metatarsal load	No	20 (33.3)	3 (10)	17 (56.7)	<0.001
	Yes	40 (66.7)	27 (90)	13 (43.3)	
Metatarsal index	Index Minus	41 (68.3)	19 (63.3)	22 (73.3)	0.028
	Index Plus	4 (6.7)	0 (0)	4 (13.3)	
	Index Plus-Minus	15 (25%)	11 (36.7)	4 (13.3)	

RIT: Reverdin–Isham translation; RI: Reverdin–Isham; MTF metatarsophalangeal. ^a^ Fisher test.

**Table 2:** Changes in clinical and radiological variables from the preoperative to the postoperative assessment in both cohorts.

	RIT Cohort (*n* = 30)	RI Cohort (*n* = 30)	*p*-Value ^c^
Pain (VAS) ^a^			
Preoperative	8.33, SD 0.84 (7–10)	7.17, SD 0.83 (5–8)	
Postoperative	1.17, SD 1.23 (0–6)	0.47, SD 0.73 (0–2)	
*p* value ^b^	<0.001	<0.001	
Difference	−7.17	−6.70	0.137
AOFAS score ^a^			
Preoperative	41.07, SD 15.49 (5–70)	51.33, SD 16.20 (5–80)	
Postoperative	88.33, SD 7.73 (73–100)	93.83, SD 9.38 (70–100)	
*p* value ^b^	<0.001	<0.001	
Difference	47.27	42.50	0.203
MPA ^a^			
Preoperative	33.40, SD 12.48 (2–54)	23.80, SD 7.84 (11–42)	
Postoperative	10.27, SD 3.62 (3–20)	10.60, SD 3.46 (3–18)	
*p* value ^b^	<0.001	<0.001	
Difference	−23.13	−13.20	<0.001
IMA ^a^			
Preoperative	17.03, SD 4.65 (10–34)	12.53, SD 2.73 (8–18)	
Postoperative	11.10, SD 2.83 (8–21)	9.23, SD 0.82 (8–11)	
*p* value ^b^	<0.001	<0.001	
Difference	−5.93	−3.30	0.001
PASA ^a^			
Preoperative	13.10, SD 6.18 (7–41)	7.50, SD 2.45 (4–14)	
Postoperative	5.87, SD 1.93 (2–9)	2.37, SD 1.40 (0–5)	
*p* value ^b^	<0.001	<0.001	
Difference	−7.23	−5.13	0.201
DASA ^a^			
Preoperative	10.87, SD 4.48 (2–19)	7.53, SD 2.97 (4–16)	
Postoperative	5.23, SD 2.31 (1–11)	2.00, SD 1.78 (0–5)	
*p* value ^b^	<0.001	<0.001	
Difference	−5.63	−5.53	0.964
Sesamoid position ^a^			
Preoperative	6.00, SD 1.34 (1–7)	2.80, SD 1.37 (1–7)	
Postoperative	1.77, SD 0.68 (1–3)	1.23, SD 0.43 (1–2)	
*p* value ^b^	<0.001	<0.001	
Difference	−4.23	−1.57	<0.001

RIT: Reverdin–Isham translation; RI: Reverdin–Isham; VAS: visual analogue scale; AOFAS: American Orthopaedic Foot and Ankle Society; MPA: metatarsophalangeal angle; IMA: intermetatarsal angle; PASA: proximal articular set angle; DASA: distal articular set angle. ^a^ Mean, SD (range) ^b^ Wilcoxon test ^c^ Mann–Whitney test.

**Table 3:** Crude and multivariate adjustment using Delta variables as dependent variables adjusted for age, pressure pain, and load transfer to central.

Raw Adjustment					Multivariate Adjustment			
		Betas	Error	*p*-Value	Betas	Error	*p*-Value	% R2
DELTA_Pain (VAS)	Intercept	−6.70	0.21	<0.001	−7.29	0.81	<0.001	0.6
	RIT	−0.47	0.31	0.135	−0.36	0.37	0.335	
DELTA_Total AOFAS score	Intercept	42.50	2.46	<0.001	33.21	8.98	<0.001	3.9
	RIT	4.77	3.48	0.177	1.05	4.06	0.795	
DELTA_MPA	Intercept	−13.20	1.68	<0.001	0.19	5.89	0.974	29.4
	RIT	−9.93	2.38	<0.001	−7.23	2.66	0.009	
DELTA_IMA	Intercept	−3.30	0.53	<0.001	−1.37	1.92	0.477	19.8
	RIT	−2.63	0.75	<0.001	−3.63	0.87	<0.001	
DELTA_PASA	Intercept	−5.13	0.95	<0.001	−6.90	3.57	0.060	2.5
	RIT	−2.10	1.34	0.124	−2.28	1.61	0.164	
DELTA_DASA	Intercept	−5.53	0.62	<0.001	−7.01	2.36	0.004	5.2
	RIT	−0.10	0.89	0.911	0.35	1.06	0.743	
DELTA_Sesamoid_Position	Intercept	−1.56	0.23	<0.001	−2.00	0.83	0.02	54.5
	RIT	−2.66	0.32	<0.001	−2.25	0.37	<0.001	

RIT: Reverdin–Isham translation; VAS: visual analogue scale; AOFAS: American Orthopaedic Foot and Ankle Society; MPA: metatarsophalangeal angle; IMA: intermetatarsal angle; PASA: proximal articular set angle; DASA: distal articular set angle.

**Table 4:** Comparison of MPA, IMA, PASA, and AOFAS score improvements between the two techniques used in the present study and those found in previous systematic reviews.

Study	Technique	MPA Improvement	IMA Improvement	PASA Improvement	AOFAS Score Improvement
Present Study	Reverdin–Isham (Standard)	13.20	3.30	5.13	42.50
	Reverdin–Isham with Lateral Translation (RIT)	23.13	5.93	7.23	47.27
Malagelada et al. [13]	Reverdin–Isham	8.6–17.1	0.9–5.2	NR	33.1–49.8
	Bösch	10–19.7	4.8–9.6	NR	30.2–53.7
Bia et al. [23]	Reverdin–Isham	9.3–15	0.2–5	NR	35.2–45
	Bösch (Percutaneous)	19.6	9.5	NR	42.2
Caravelli et al. [8]	Bösch	16.5	6	NR	20
	Reverdin–Isham	14	3	NR	20
Trnka et al. [24]	Bösch	17–20.9	3–3.5	NR	NR
	Reverdin–Isham	18.9	4	NR	41

MPA: metatarsophalangeal angle; IMA: intermetatarsal angle; PASA: proximal articular set angle; AOFAS: American Orthopaedic Foot & Ankle Society Score; NR: not reported.

## Data Availability

The data presented in this study are available on request from the corresponding authors due to privacy reasons.

## Supplementary Materials

The following supporting information can be downloaded at: https://www.mdpi.com/article/10.3390/jcm13185468/s1, Figure S1: Intraoperative fluoroscopy with the surgeon performing the release of soft tissues; Figure S2: Intraoperative fluoroscopy with displacement of the metatarsal head assisted by the surgeon; Figure S3: Displacement of the metatarsal head assisted by the surgeon after performing the Reverdin–Isham osteotomy with lateral translation; Figure S4: Frontal view of the right foot with severe hallux valgus; Figure S5: Weight-bearing anteroposterior X-ray of the right foot with severe hallux valgus; Figure S6: Intraoperative fluoroscopy of severe hallux valgus correction; Figure S7: Anteroposterior view and weight-bearing anteroposterior X-ray of the right foot with moderate hallux valgus from a patient who underwent a modified Reverdin–Isham osteotomy with lateral translation, (**a**) before and (**b**) at 6 months after surgery; Figure S8: Intraoperative fluoroscopy with hypercorrection of the first ray position; Figure S9: Bandage after Reverdin–Isham osteotomy with lateral translation of the first metatarsal head; Table S1: AOFAS domains corresponding to the two cohorts before and after surgery; Table S2: Analysis by delta variables; Table S3: Normality analysis of the Delta variables.
