# Supplementary material for: Comparison of the Minimally Invasive Reverdin–Isham Lateral Translation Osteotomy Versus the Standard Reverdin–Isham Technique: A Pilot Prospective Cohort Study
Source: J Clin Med. 2024 Sep 14;13(18):5468. doi: 10.3390/jcm13185468 (PMC11432747; doi:10.3390/jcm13185468)
Supplement: Supplementary file 1 [file jcm-13-05468-s001.zip › jcm-3162389-Supplementary tables.pdf]

Supplementary Table S1. AOFAS domains corresponding to the two cohorts before and after surgery.

Cohort	AOFAS domain	n	Mean	SD	95%CI	p-value[a]
RIT	Pain (pre)	30	13.67	9.99	(10.00-17.00)	<0.001
	Pain (post)	30	35.00	5.72	(33.00-37.00)	
	Foot functionality (pre)	30	4.83	1.95	(4.10-5.47)	<0.001
	Foot functionality (post)	30	8.90	1.47	(8.30-9.40)	
	Footwear (pre)	30	4.83	2.45	(4.00-5.67)	<0.001
	Footwear (post)	30	7.00	2.49	(6.17-7.83)	
	Mobility AMFH (pre)	30	5.83	2.96	(4.67-6.83)	<0.001
	Mobility AMFH (post)	30	8.83	2.15	(8.00-9.50)	
	Mobility IF (pre)	30	4.83	0.91	(4.50-5.00)	1.000
	Mobility IF (post)	30	4.83	0.91	(4.50-5.00)	
	Stability AMF-IF (pre)	30	4.00	2.03	(3.17-4.67)	0.014
	Stability AMF-IF (post)	30	5.00	0.00	(5.00-5.00)	
	Callus MTF-IF (pre)	30	1.50	2.33	(0.67-2.33)	<0.001
	Callus MTF-IF (post)	30	5.00	0.00	(5.00-5.00)	
	Alignment (pre)	30	1.07	2.77	(0.27-2.13)	<0.001
	Alignment (post)	30	13.60	2.85	(12.67-14.53)	
	Pain (pre)	30	21.67	5.92	(19.68-23.67)	<0.001
	Pain (post)	30	37.33	4.50	(35.67-38.67)	
	Foot functionality (pre)	30	6.07	2.33	(5.27-6.87)	<0.001
	Foot functionality (post)	30	9.70	0.92	(9.30-10.00)	
	Footwear (pre)	30	5.17	2.07	(4.50-5.83)	<0.001
	Footwear (post)	30	8.00	2.82	(7.00-9.00)	
	Mobility AMFH (pre)	30	6.00	2.75	(5.00-7.00)	<0.001
	Mobility AMFH (post)	30	10.00	0.00	(10.00-10.00)	
	Mobility IF (pre)	30	3.83	2.15	(3.00-4.50)	0.008
	Mobility IF (post)	30	5.00	0.00	(5.00-5.00)	
	Stability AMF-IF (pre)	30	4.17	1.90	(3.50-4.67)	0.025
	Stability AMF-IF (post)	30	5.00	0.00	(5.00-5.00)	
	Callus MTF-IF (pre)	30	0.33	1.27	(0.00-0.83)	<0.001
	Callus MTF-IF (post)	30	4.83	0.91	(4.50-5.00)	
	Alignment (pre)	30	4.77	4.42	(3.20-6.30)	<0.001
	Alignment (post)	30	14.30	2.14	(13.60-15.00)	

SD: Standard deviation; 95%CI: 95% confidence interval; AMFH: Anatomical Metatarsal-First Hallux; IF: Interphalangeal Joint; AMF-IF: Anatomical Metatarsal-First Interphalangeal Joint; MTF-IF: Metatarsophalangeal-First Interphalangeal Joint; pre: preoperative; post: postoperative

[a]Wilcoxon test

Supplementary Table S2. Analysis by delta variables.

		n	Mean	SD	p-value[a]
DELTA_Pain (EVA)	RIT	30	-7.17	1.42	0.137
	RI	30	-6.70	0.92	
DELTA_Total AOFAS	RIT	30	47.27	13.72	0.203
	RI	30	42.50	13.28	
DELTA_MPA	RIT	30	-23.13	11.47	<0.001
	RI	30	-13.20	6.17	
DELTA_IM	RIT	30	-5.93	3.14	0.001
	RI	30	-3.30	2.69	
DELTA_PASA	RIT	30	-7.23	6.96	0.201
	RI	30	-5.13	2.39	
DELTA_DASA	RIT	30	-5.63	4.11	0.964
	RI	30	-5.53	2.62	
DELTA_Sesamoid_Position	RIT	30	-4.23	1.28	<0.001
	RI	30	-1.57	1.22	

SD: standard deviation; MPA: metatarsophalangeal angle; IMA: intermetatarsal angle; PASA: proximal articular set angle; DASA: distal articular set angle

[a]Mann-Whitney test

Supplementary Table S3. Normality analysis of the Delta variables.

	Cohort	p-value[a]
DELTA_Pain (EVA)	RIT	0.284
	RI	0.011
DELTA_Total AOFAS	RIT	0.881
	RI	0.733
DELTA_MPA	RIT	0.390
	RI	0.542
DELTA_IM	RIT	0.657
	RI	0.044
DELTA_PASA	RIT	0.077
	RI	0.532
DELTA_DASA	RIT	0.772
	RI	0.199
DELTA_Sesamoid_Position	RIT	0.089
	RI	0.205

MPA: metatarsophalangeal angle; IMA: intermetatarsal angle; PASA: proximal articular set angle; DASA: distal articular set angle

[a]Kolmogorov-Smirnov test
