# Supplementary figures and images for: Comparison of the Minimally Invasive Reverdin–Isham Lateral Translation Osteotomy Versus the Standard Reverdin–Isham Technique: A Pilot Prospective Cohort Study
Source: J Clin Med. 2024 Sep 14;13(18):5468. doi: 10.3390/jcm13185468 (PMC11432747; doi:10.3390/jcm13185468)

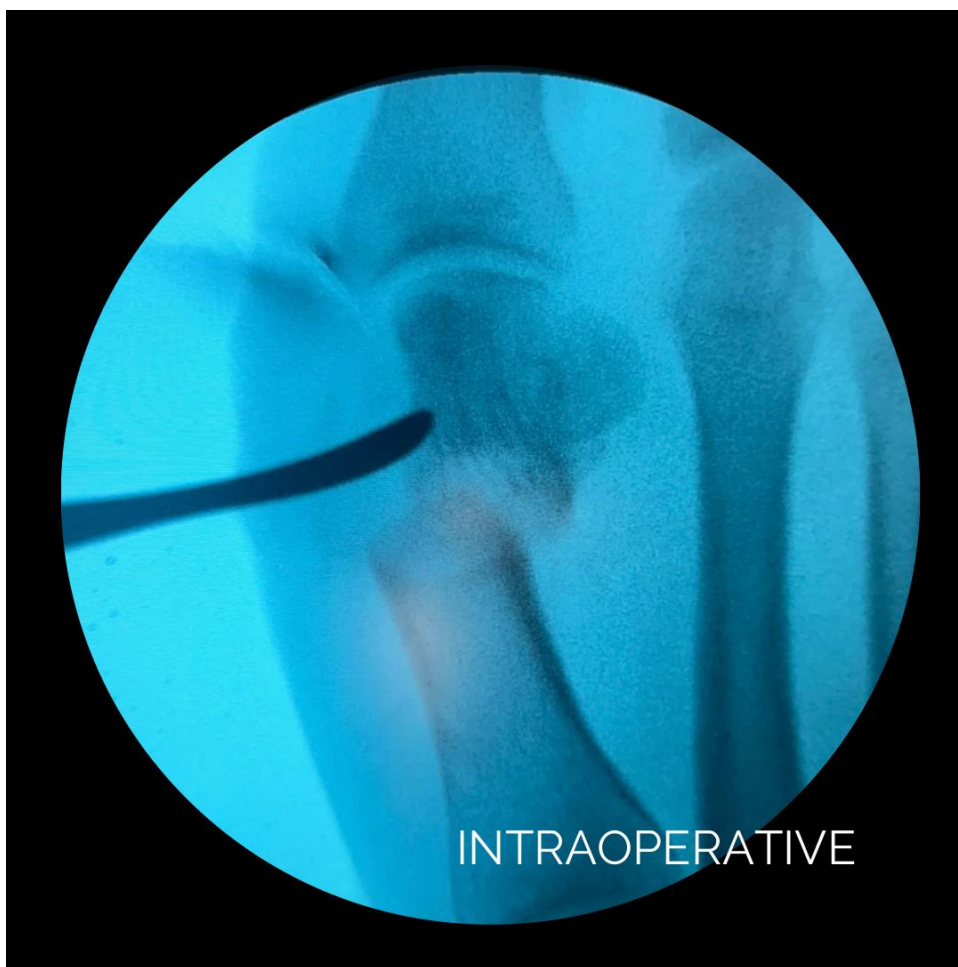

**Figure S1**

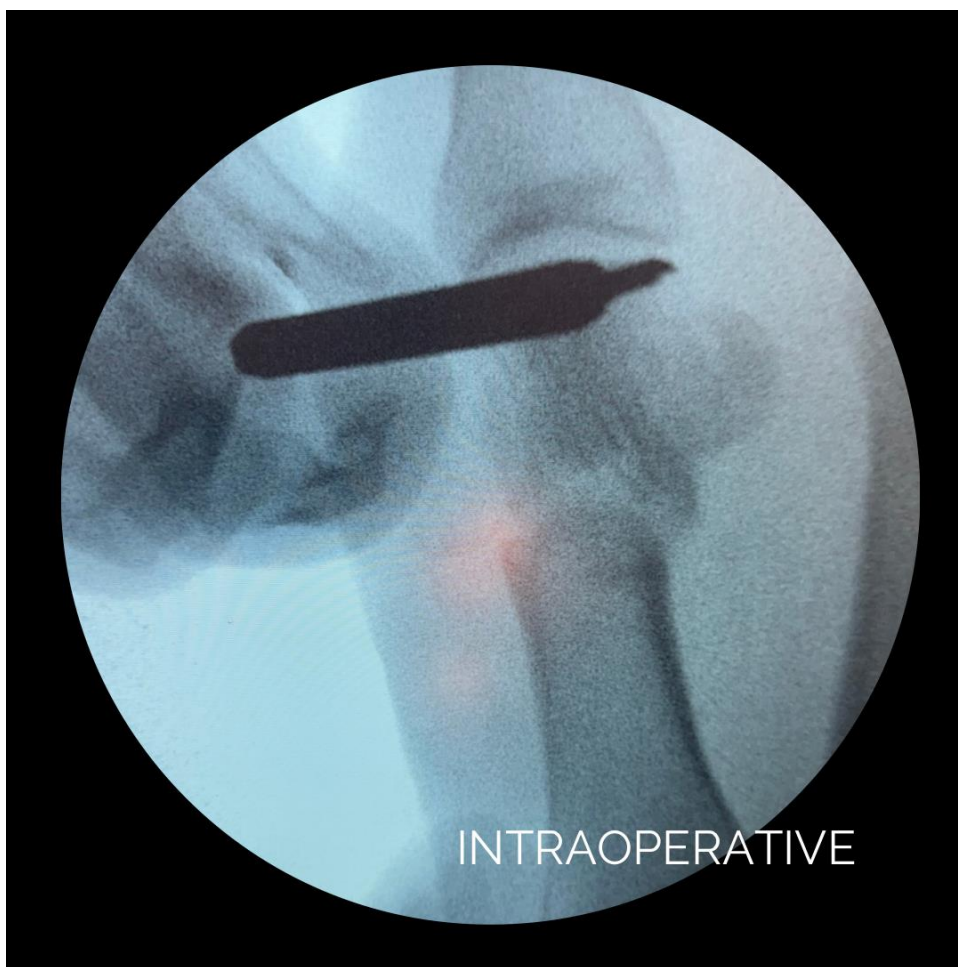

Figure S2

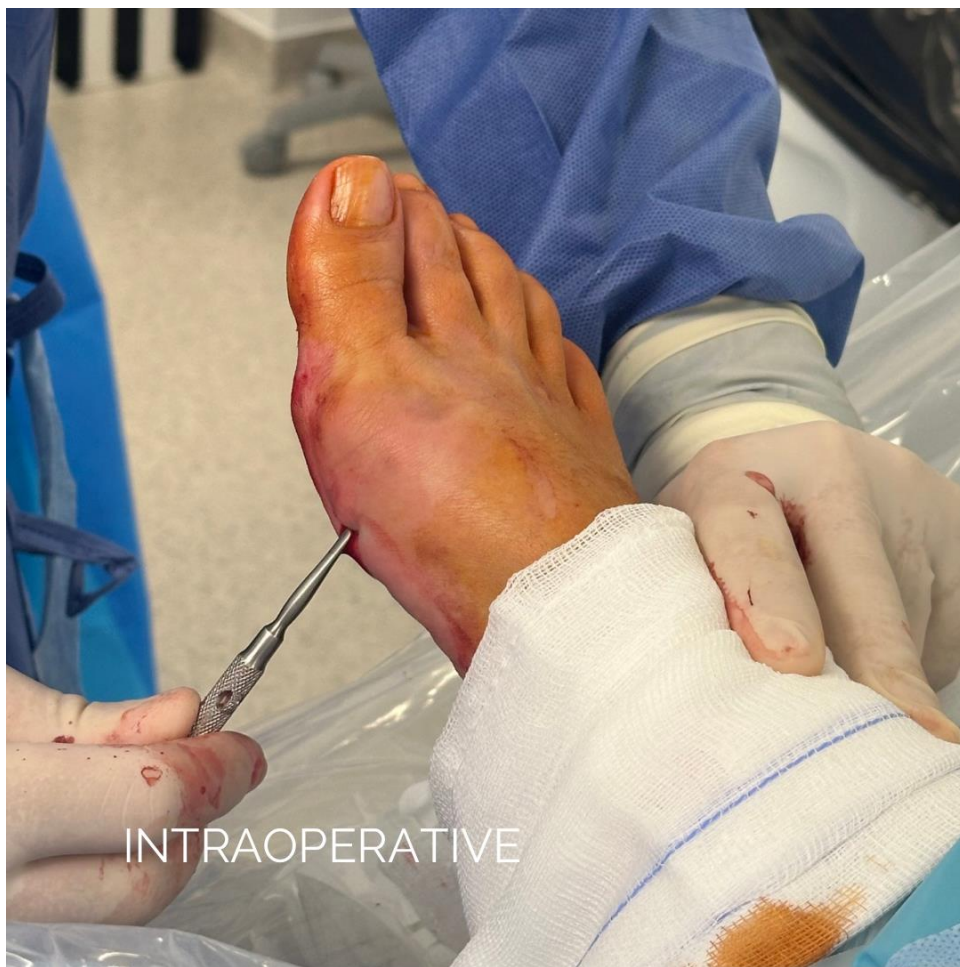

**Figure S3**

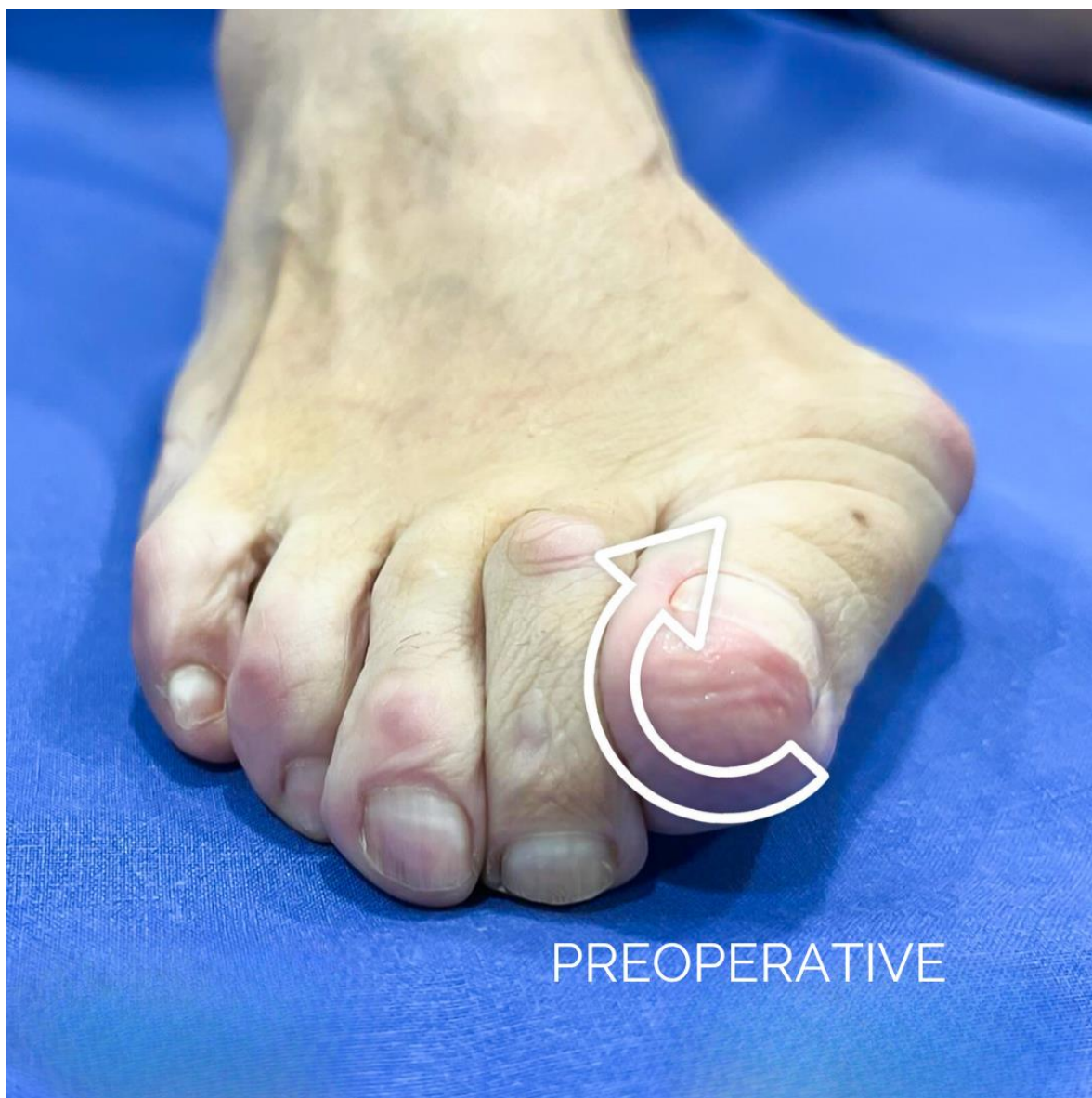

**Figure S4**

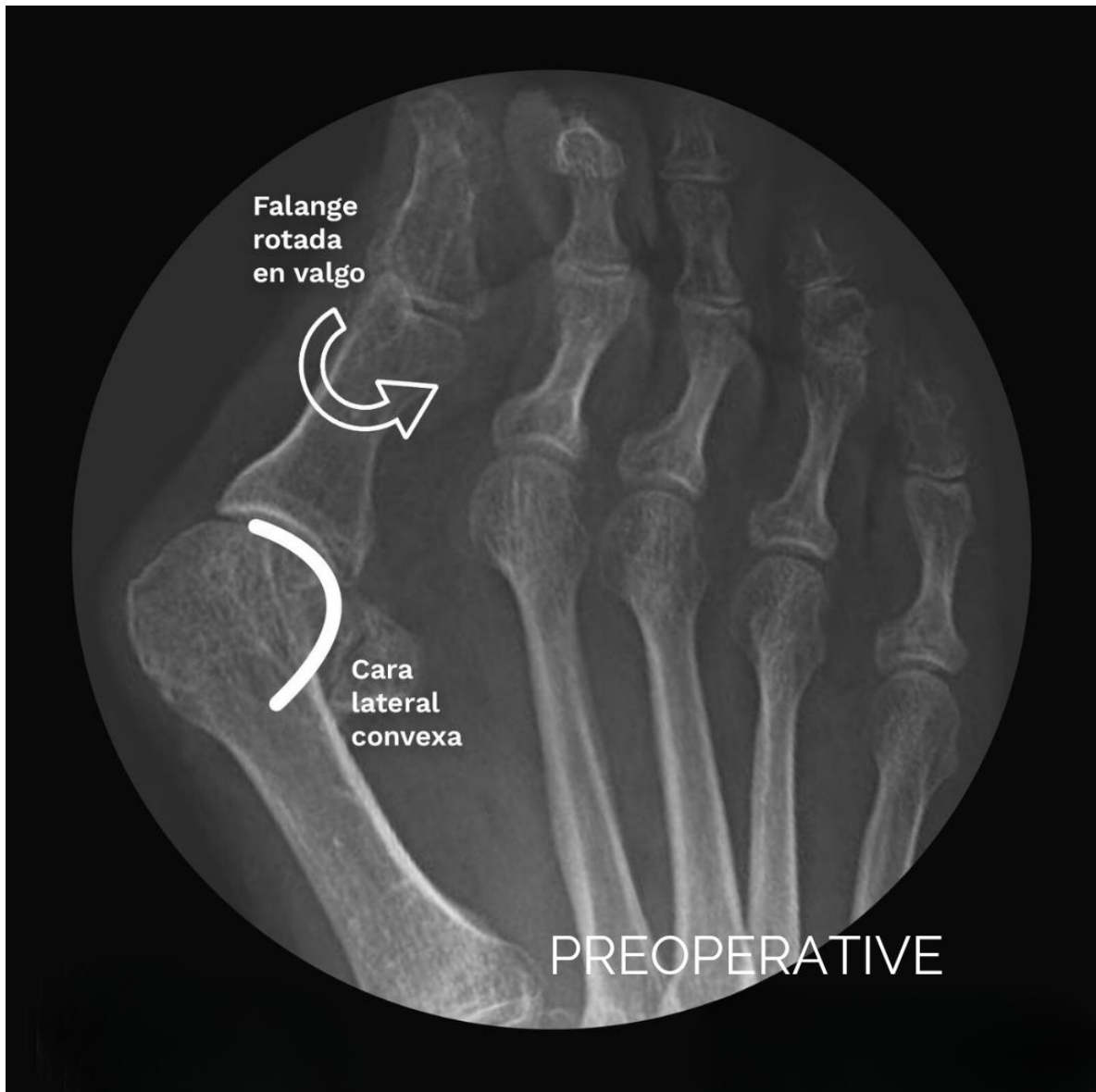

Figure S5

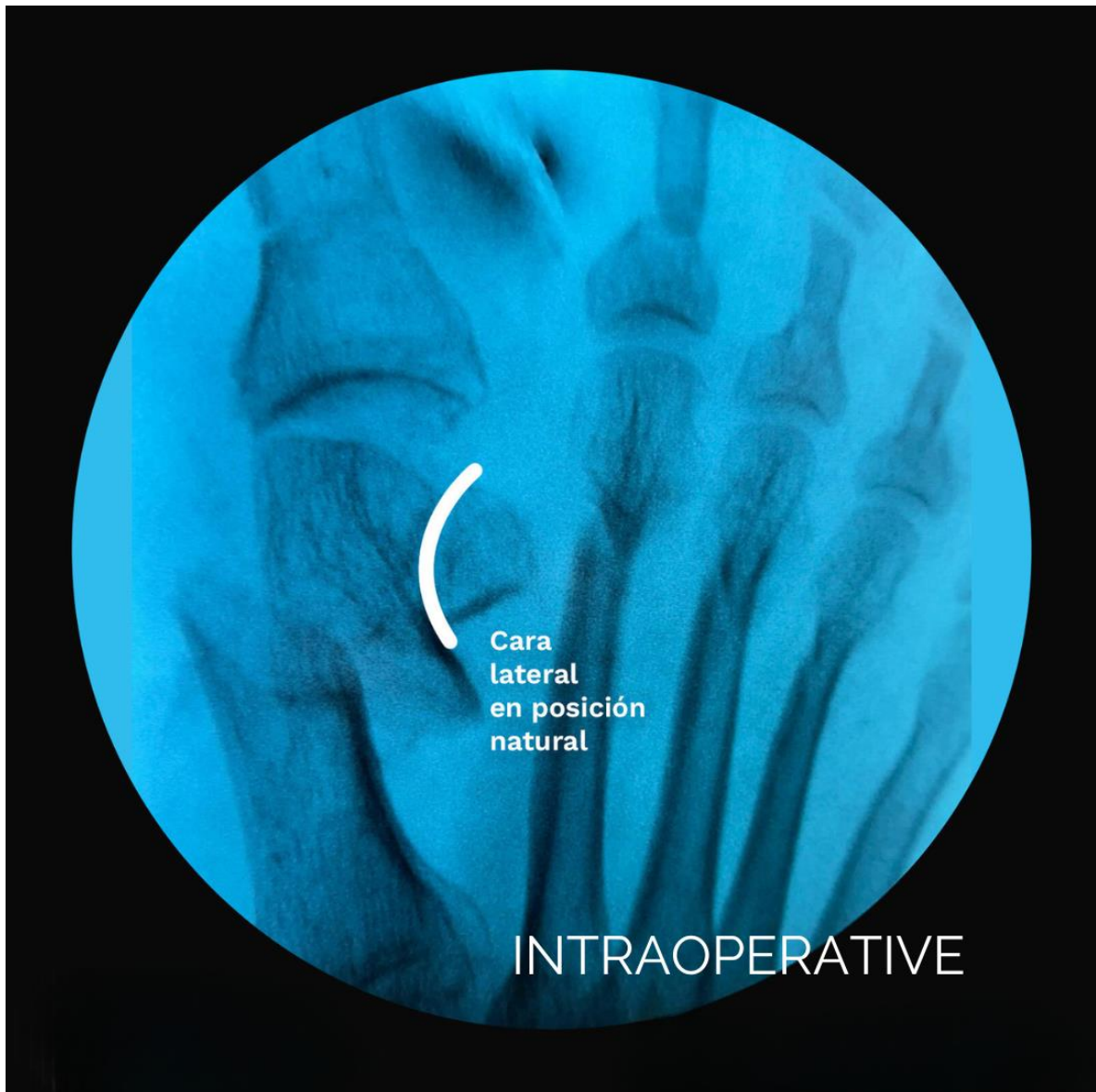

Figure S6

7a

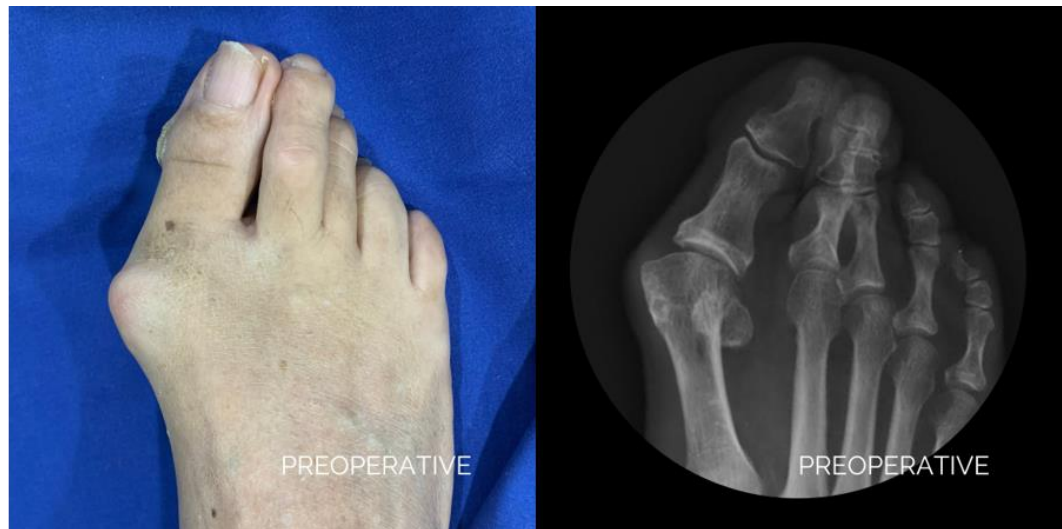

7b

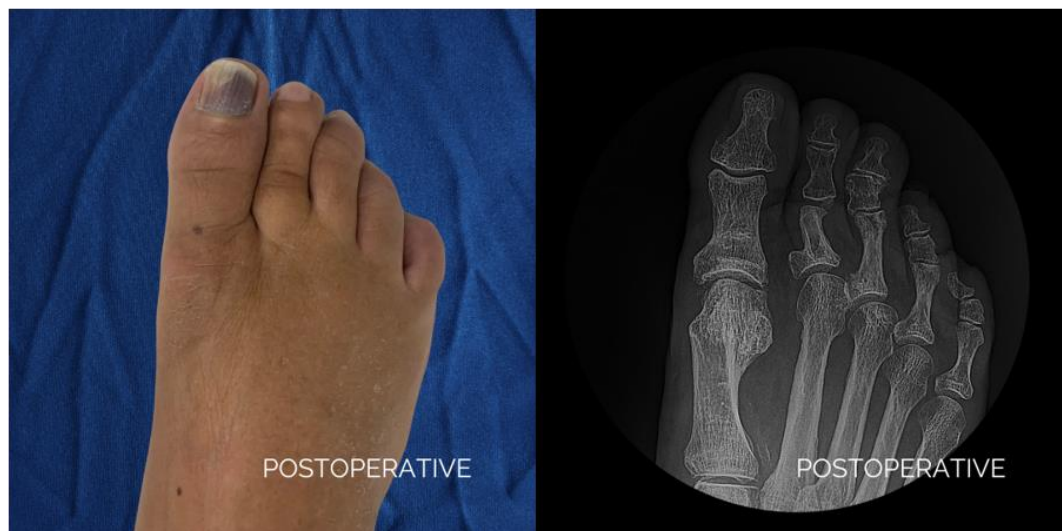

Figure S7

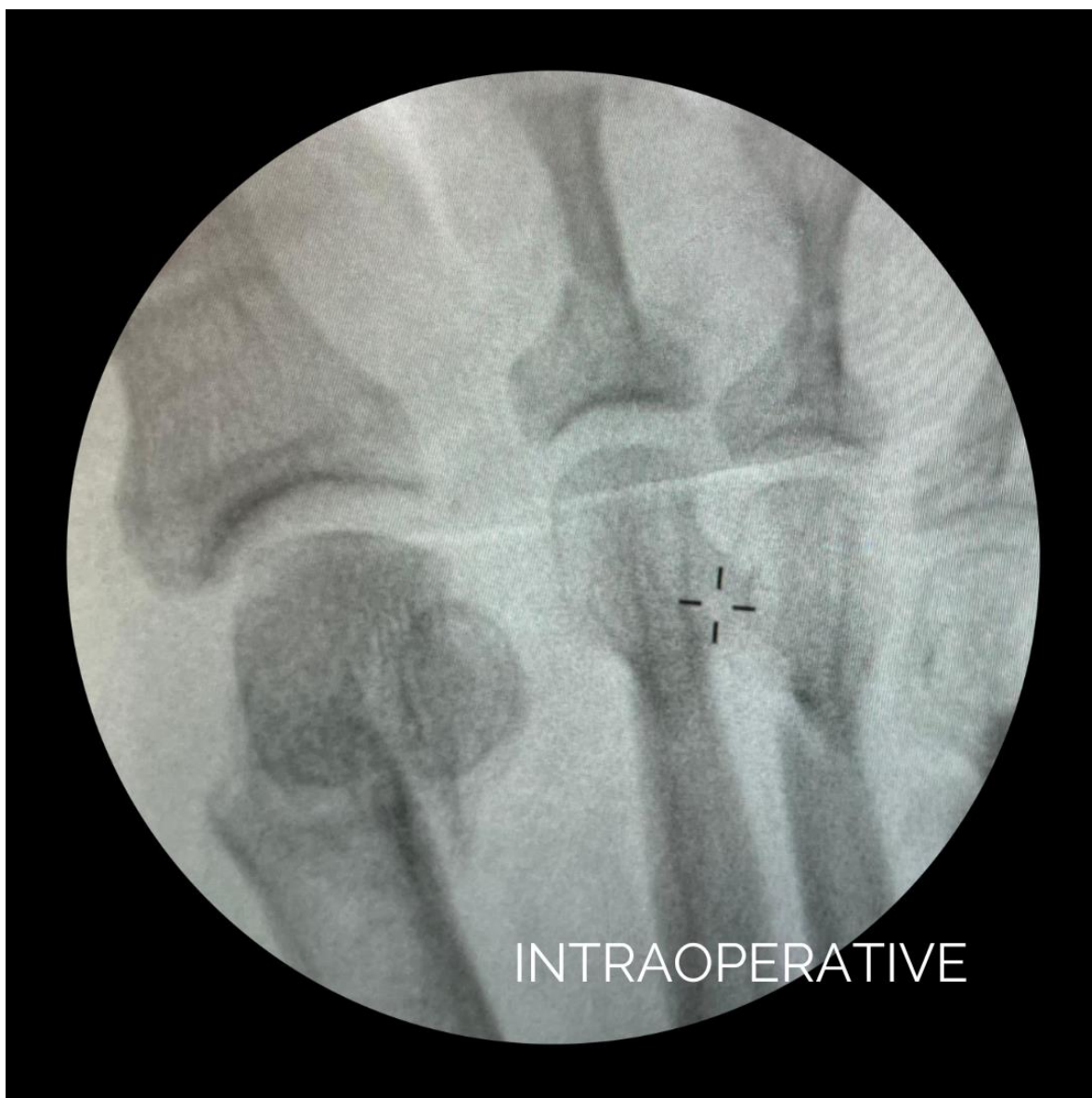

Figure S8

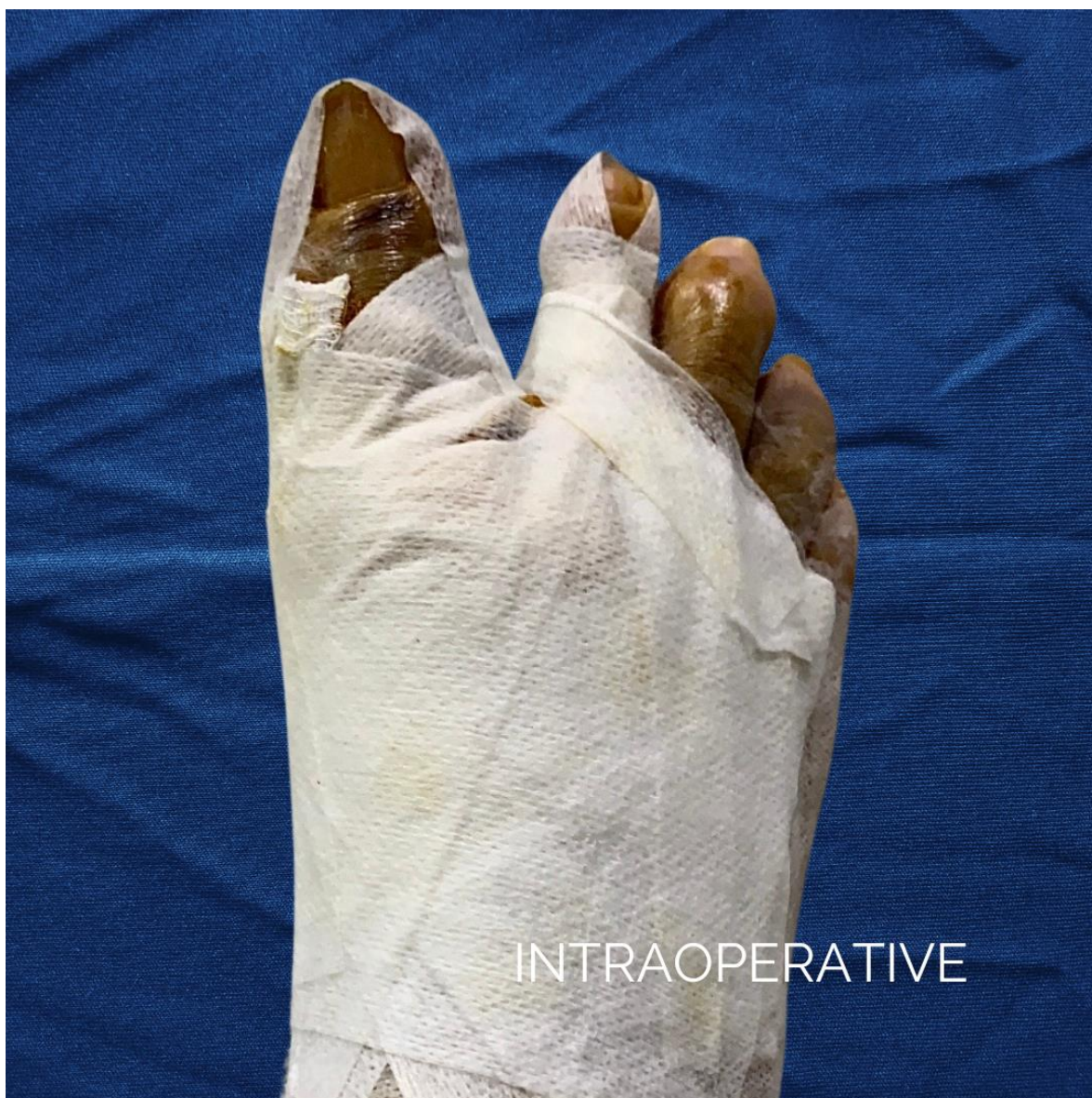

Figure S9

Supplement: Supplementary file 1 [file jcm-13-05468-s001.zip › jcm-3162389-supplementary_figures.pdf]
